# To tele- or not to telehealth? Ongoing COVID-19 challenges for private psychiatry in Australia

**DOI:** 10.1177/1039856220950081

**Published:** 2020-10

**Authors:** Jeffrey CL Looi, William Pring

**Affiliations:** Academic Unit of Psychiatry and Addiction Medicine, Australian National University Medical School, Canberra Hospital, Australia; Private Psychiatry, Australia; Monash University, and Centre for Mental Health Education and Research at Delmont Private Hospital, Australia; Private Psychiatry, Australia

**Keywords:** telehealth, telepsychiatry, private practice, COVID-19, psychiatrist

## Abstract

**Objectives:**

Following a very rapid and significant uptake of metropolitan telepsychiatry in private practice in Australia during COVID-19, practical questions remain: How long should psychiatrists continue telepsychiatry? Are there benefits of continuing: reduced COVID-19 risks to patient and psychiatrist, and flexibility of appointments? Will the Medicare Benefits Schedule (MBS) telehealth items be retained? How does metropolitan telepsychiatry fit into the overall mix of public and private services? This is an important debate.

**Conclusions:**

Private psychiatrists may continue to offer the majority of care, where practical, via telepsychiatry to reduce COVID-19 exposure risks, as well as allow for the realities of practice management for pandemic public health measures. However, consideration has to be given to the potential drawbacks for patients with sight, hearing and illness-related disabilities or risks, when in-person consultation is required. There are also risks: some patients may not benefit from telepsychiatry due to the nature of their illness, and will patients feel rapport is lost? However, the retention of COVID-19 MBS telehealth items is needed for ongoing flexible and comprehensive private practice psychiatry.

In the context of the COVID-19 pandemic, there has been a rapid shift to provision of telehealth in general,^1^ and private telepsychiatry, mediated by the new Medicare Benefits Schedule (MBS) telehealth items.^2^ Absent the development of a vaccine for COVID-19, social distancing and hygiene measures remain in place in Australia in mid-August 2020, with Victoria introducing Stage 4 distancing restrictions to control the second wave of infections.^3^ While direct personal protective equipment (PPE) may not be required unless psychiatrists themselves are vulnerable, it is prudent to consider how private psychiatric practice may continue while also reducing the risk of COVID-19 and other community-borne infections.

## Benefits of ongoing telepsychiatry

Among the benefits of ongoing telepsychiatry provision is the maintenance of COVID-19 social distancing and hygiene for patients and psychiatrists. This also includes avoidance of the need for screening of patients for COVID-19 symptoms for attendance, cleaning of the consultation room, as well as the potential awkwardness of maintaining distancing in the room (e.g. having markings on the floor to exclude patients from approaching the psychiatrist or *vice versa*, or use of a Perspex screen).

Maintenance of patient care continuity has been an advantage of telepsychiatry, in that patients have been able to access consultation, whatever the stage of COVID-19 measures, at least via telephone. Practically, telepsychiatry has allowed for greater flexibility in scheduling appointments, as the requirement for patients to attend in-person is obviated, and accordingly appointments can more easily be fitted around mutual convenience. In addition, for private outpatient practice, telepsychiatry has helped ensure business continuity through ongoing consultations, when general practice^4^ and other medical specialties have seen a decline in attendance via telehealth or in person. However, it is acknowledged that the phased introduction of the MBS telehealth item numbers, as well as initial lack of items for new patient consultations, gradual reduction of bulk-billing restrictions, and other aspects, hampered implementation.

There is emerging anecdotal evidence that certain patient populations may prefer telepsychiatry. Perinatal mental health services have noted a rapid uptake and comfort with telepsychiatry by women, especially those with young children. Elderly veterans, with comorbid health conditions increasing their risk for COVID-19, have been comfortable with telephone telehealth.

Telepsychiatry affords the opportunity to provide enhanced care for the follow-up of patient enquiries, requests for prescriptions, correspondence and the results of investigations; in that psychiatrists can video or teleconference with patients to understand, and explain and provide psychiatric advice in regard to such enquiries using the MBS telehealth items. Remuneration of these items is likely now to capture a view of a small fraction of the extensive, previously *pro bono* work performed by psychiatrists in day-to-day care of patients. The ongoing provision of appropriate remuneration, via MBS telehealth items for such work will improve the clinical and business sustainability of private practices, thus overall strengthening the resilience of private psychiatry and the complementarity with public mental health services.

The implementation of COVID-19 measures for prescriptions via telehealth was meant to improve the effectiveness of the provision of medication through enabling of electronic prescribing (e-prescribing) to allow provision of medication through a patient’s pharmacy.^5^ E-prescribing, in conjunction with a telehealth MBS item involves a paper prescription which can be transmitted digitally (email, text or fax) to the patient’s pharmacist.^5^ Generally, full e-prescribing was not uniformly available for psychiatrists during COVID-19 measures, and there remained implementation problems. Faxing or emailing prescription images was not accepted by all pharmacists. The consequent mailing of prescriptions resulted in up to 2 weeks of delay or loss of prescriptions, due to COVID-19 measures affecting postal services. This necessitated more psychiatrist contacts with patients to ensure supply of medications. However, it is hoped that continued e-prescribing will expedite telepsychiatry practice and assist in maintaining social distancing and hygiene by removing the need for patients to provide a paper prescription to their pharmacist. E-prescribing effectiveness will also need to be evaluated.

While the existing evidence base for mental health telehealth provision is that patients and practitioners find it practical and effective,^6^ specific evaluation of metropolitan private telepsychiatry is needed in terms of outcomes, patient and psychiatrist satisfaction, as well as health economic implications.^2^ Similarly, the technology of telepsychiatry, including cybersecurity, will also require evaluation. From such evaluations, standards and accreditation for safety and quality control may be developed.

## Risks and challenges of ongoing telepsychiatry

There needs to be ongoing careful consideration as to whether telepsychiatry is fit for purpose for patients and their needs. Video consultation may be more effective in developing and maintaining rapport, given the preservation of a modicum of visual cues and body language. Telephone consultation, with minimal non-verbal cues, may be more suitable for patients with established therapeutic relationships. Conveying empathy via video or telephone may be more challenging, and this likely disproportionately affects psychotherapy provision. While telehealth may be perceived as less empathic and engaging, our recent rapid review has found telehealth for mental health provision is indeed effective.^6^ Nonetheless, there is an argument that some new patients should be seen in person to better establish rapport, with appropriate social distancing. Similarly, patients with sight and hearing impairments, or with intellectual disabilities, cognitive deficits and specific mental health risks may also need to be seen in person. The preference of patients for in-person consultation is also important and needs to be seriously considered, against a background that most patients are not seen in person to reduce overall risk for patients and psychiatrists. A safety plan needs to be negotiated by the psychiatrist with the patient who agrees to telepsychiatry, based on screening at the time of scheduling appointments by trained practice staff, with ready back-up from the psychiatrist.

Overall, though telehealth for mental health care provision has been demonstrated to be effective across the lifespan,^6^ there remain some limitations in using telepsychiatry. There should be screening as to suitability for telepsychiatry. Assessments that require detailed observation and/or physical examination or patients with disabilities not suitable for video or telephony are not suitable for telepsychiatry. Similarly, patients with significant issues of risk of harm may not be suitable or may require implementing a specific safety plan. Patients with psychotic illnesses may not be comfortable with video or telephone telepsychiatry. Some children and adolescents may prefer communicating via text messages or online text-based media. There is also the risk of reduced access for patients with socioeconomic disadvantage due to lack of technological literacy, telephony or internet connectivity.

There is a risk that telehealth-only consultation, with no or very limited options for in-person consultation, may unduly constrain comprehensive care, for some of the reasons identified above. However, there have been some established telehealth services (e.g. https://telepsychiatrist.online/) for rural and remote psychiatry that function entirely by tele or videoconferencing. Similarly, during COVID-19, some completely online psychiatrist (combined with other specialists) telehealth services have emerged (e.g. https://dokotela.com.au/). The roles, benefits and risks of largely corporatised telepsychiatry services should be evaluated.

## Can ongoing telepsychiatry work with existing private mental health services?

Given the evolving COVID-19 pandemic, it remains to be determined for how long a proportion of private psychiatric care will be provided by telepsychiatry. A key determinant is whether the Commonwealth Government continues the new metropolitan MBS telehealth item numbers. The pandemic status of COVID-19 has seen more restrictive social distancing and hygiene measures reinstituted with a second wave of infection in Victoria.^3^ Adaptation to provision of psychiatric care via telepsychiatry is in progress and will likely continue, analogous to the adaptations the public has made regarding social distancing for work and home life during COVID-19. Accordingly, telepsychiatry may become a normal part of comprehensive psychiatric practice, despite the limitations above; while provisions will still be needed for in-person care for patients for which telepsychiatry is not preferred or suitable.

For private psychiatric patients requiring inpatient care, there are no MBS telehealth items for psychiatric consultation. While this might seem intuitive, i.e. that patients requiring inpatient care have more severe illness requiring in-person care and consultation, there remains the possibility that, as a scarce specialist resource, psychiatrists may either not be available (depending on the infection status of the COVID-19 pandemic) or individual psychiatrists might be vulnerable to COVID-19 (age, comorbid illness, etc.) On this basis, consideration should be given to possible specific conditional options for telepsychiatry for psychiatric inpatients, including for the provision of second/further opinions from other psychiatrists to provide advice for care.

The provision of individual and group psychotherapy via telehealth – while effective on evidence base^6^ – has anecdotally been found by psychotherapy-focused psychiatrists to be less useful in practice. However, access to telehealth for psychotherapy should still be retained for flexibility of appointments post-COVID-19.

Practically, in-person consultation must necessarily be retained as the gold standard for psychiatric consultation across public and private sectors on the basis of the interpersonal richness of such interaction.^2^ It is therefore likely that, given a choice, free of the need for COVID-19 pandemic public health measures, the majority of patients and psychiatrists will prefer in-person consultation. For the not too foreseeable COVID-19 pandemic future in August 2020, there may be a gradual pivot back to in-person consultations as social distancing and hygiene measures are relaxed, or conversely, increased telepsychiatry for a sustained second wave of COVID-19 infection as in Victoria. Depending on patient and psychiatrist preferences, and individual requirements for patients who are geographically isolated or suffering disabilities, the retention of access to MBS telehealth items is necessary to support telepsychiatry care, but will also permit flexibility of care provision during and post-COVID-19.

## Conclusions

While it is reasonable to continue the option of telepsychiatry provision indefinitely during and after COVID-19, it remains an individualised decision as to whether telepsychiatry is appropriate for a patient. In-person psychiatric consultation will still be likely the preferred mode of practice, but the retention of MBS telehealth items enhances the flexibility and comprehensiveness of private psychiatric practice. For private psychiatric inpatients, consideration should be given to conditional telepsychiatry consultations, at least during the COVID-19 pandemic.
